# Supplementary material for: Quantum Dot Photoluminescence Enhancement in GaAs Nanopillar Oligomers Driven by Collective Magnetic Modes
Source: Nanomaterials (Basel). 2023 Jan 27;13(3):507. doi: 10.3390/nano13030507 (PMC9919544; doi:10.3390/nano13030507)
Supplement: Supplementary file 1 [file nanomaterials-13-00507-s001.zip › nanomaterials-2164338-supplementary.pdf]

Supplementary Information: Quantum Dot Photoluminescence Enhancement in GaAs Nanopillar Oligomers Driven by Collective Magnetic Modes

Maria K. Kroychuk,[†] Alexander S. Shorokhov,[†] Damir F. Yagudin,[†] Maxim V. Rakhlin,[‡] Grigory V. Klimko,[‡] Alexey A. Toropov,[‡] Tatiana V. Shubina,[‡] and Andrey A. Fedyanin [*,†]

†*Faculty of Physics, Lomonosov Moscow State University, Moscow 119991, Russia*

‡*Ioffe Institute, 194021 St. Petersburg, Russia*

E-mail: fedyanin@nanolab.phys.msu.ru

Section S1: QDs PL analysis μ-PL spectrum of the structure schematically shown in Figure S1 is presented in Figure S2(a). QDs were excited by laser radiation with 404 nm central wavelength and signal was collected from the area of 2-3 μm. The dependence demonstrates two separate peaks which can be attributed to the emission of the QDs (from 890 nm to 1000 nm) and a wetting layer (from 880 nm to 870 nm) which is always formed for the used growth technology. When the layered structure was patterned using electron beam lithography method in the form of isolated quadrumers, the InAs QDs emission spectrum changed. The μ-PL spectrum measured at 8 K and 690 nm pump wavelength reveals a number of relatively narrow lines Figure S3(a). For a nonresonant sample, in terms of the main article, the PL intensity is lower then for resonant one, but the lines are preserved S3(b).

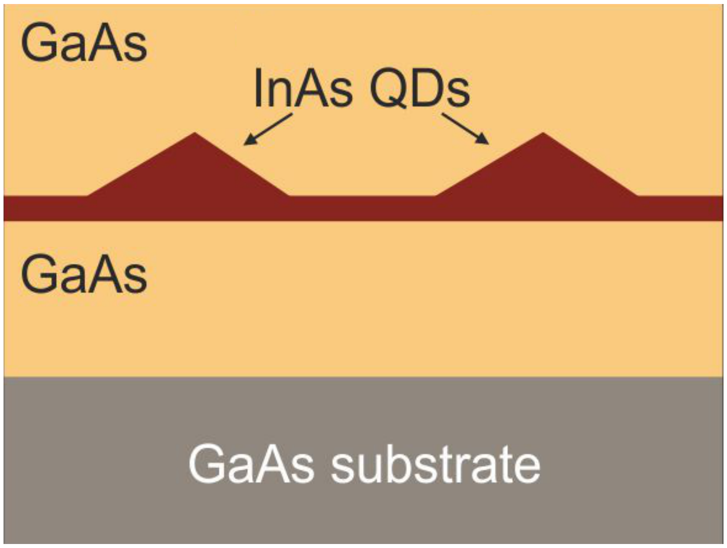

Figure S1: Schematic representation of heterostructure z-cross section consisting InAs QDs obtained using the Stranski-Krastanov growth mode in a GaAs layer on a GaAs substrate.

They are situated between 900 and 1000 nm that is similar to the emission of unstructured sample. For additional characterization of QDs, we measured their PL spectra for various pump powers and fixed excitation wavelength, 660 nm, Figure S2(b). For low powers exists one peak in the spectra associated with emission from the first excited level of a two-level system. When pump power increases another peak appears that indicates radiation from the next excited level.

Mie-resonant nanoparticle oligomers act as nanoantennas for QDs PL signal. The QDs emission looses the axial symmetry (which is typical for a source located in an unstructured volume) and is strongly modified by the radiation pattern of the magnetic dipole Mie-type resonance (MDR) of quadrumer now also showing emission along the radiation direction, which is detected in the experiment by the objective lens. Changing the nanopillar diameter one varies the MDR spectral position and reaches the case when QDs emission wavelength coincides with Mie-type mode. When the MDR spectral position approaches the QDs PL wavelength its integral measured intensity increases (Fig.3d, main text) for the system excitation in over-barrier regime ($\lambda_{pump} = 404$ nm). The experiment was realized using the μ-PL

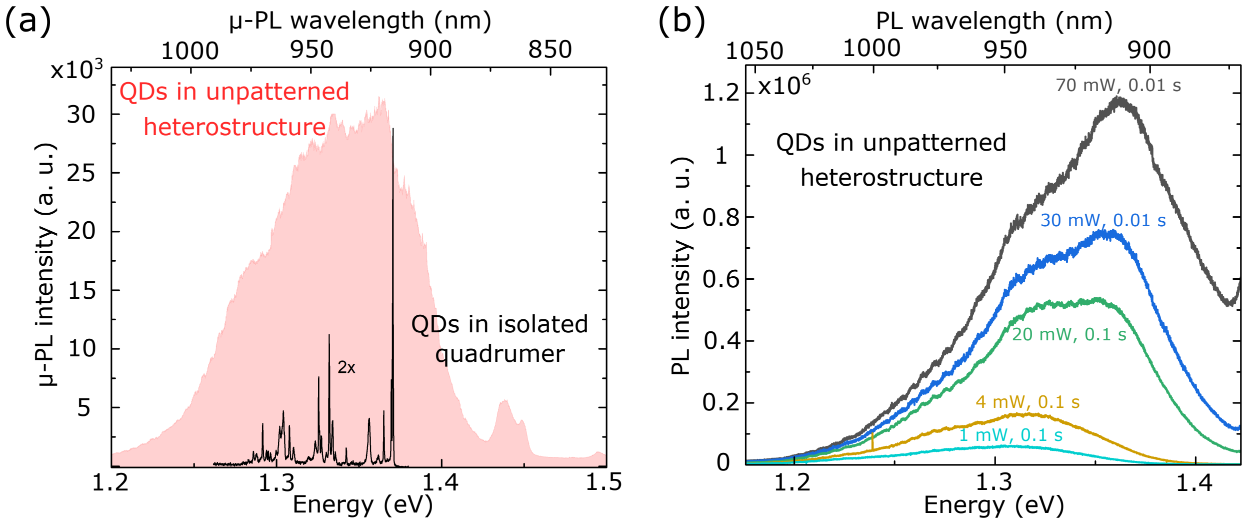

Figure S2: (a) Red area – μ-PL spectrum of InAs QDs embedded to GaAs film that is obtained from 2.5 μm spot of the sample pumped by laser radiation with 404 nm wavelength. Black curve – representative spectrum of InAs QDs located in the volume of GaAs nanopillars of isolated quadrumer demonstrated sets of emission lines. (b) PL spectra of QDs inside unstructured medium that is obtained from 200 μm spot of the sample for various pump intensities and integration times.

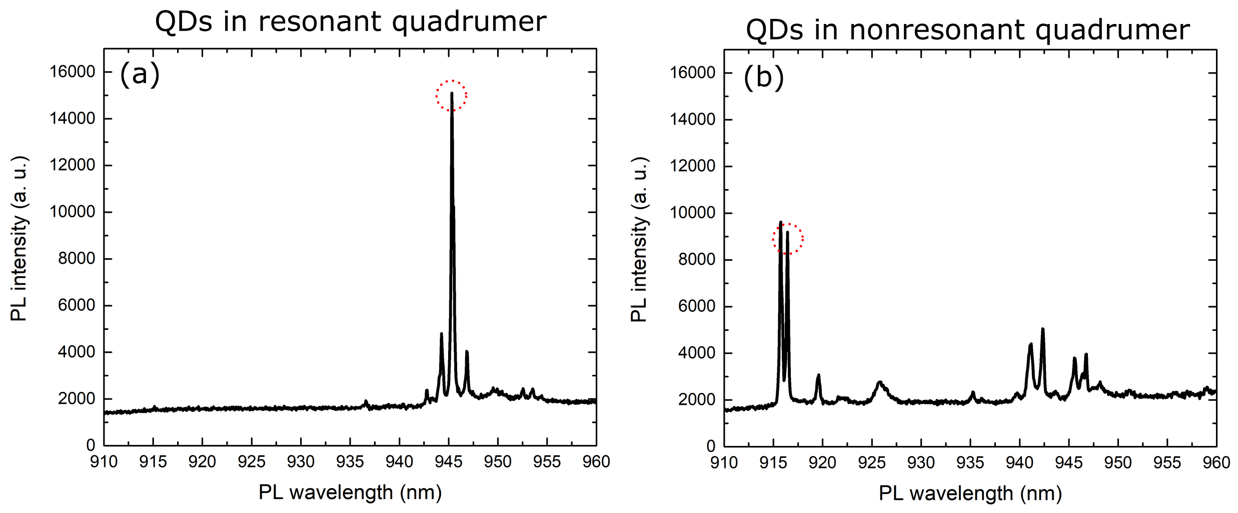

Figure S3: Representative spectra of InAs QDs located in the volume of GaAs nanopillars of isolated resonant (a) and nonresonant (b) quadrumers pumped with $\lambda = 750$ nm laser. Red circles indicate emission lines which intensities were analyzed while varying pump wavelength.

setup fully described in the main part of the paper. However, in this case we illuminated several at ones oligomers from the setup and repeated the procedure for at least 5 spatial points in the array for each diameter of nanopillar. We averaged the PL intensity over all

experimental sets for fixed diameter and found the average value of intensity in the obtained spectrum which dependence from the diameter we visualized in Figure 3d, main text.

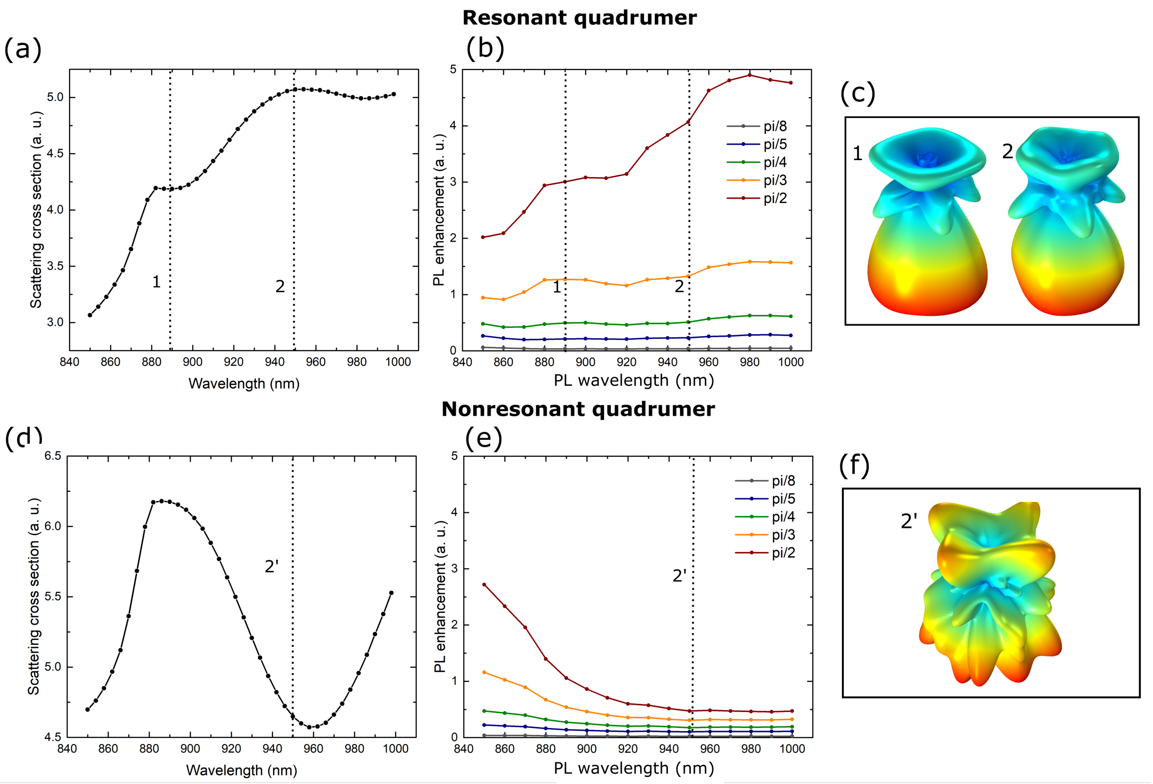

Figure S4: Scattering cross section spectra of resonant quadrumer with d = 208 nm, h = 320 nm (a) and nonresonant quadrumer with d = 300 nm, h = 320 nm (d). PL signal integrated from different solid angles for resonant quadrumer (b) and nonresonant quadrumer (e). (c), (f) dipole radiation digram embedded in resonant and nonresonant quadrumer, respectively.

We confirm that quadrumers act as nanoantennas by numerical modelling. Two types of systems are considered: resonant quadrumer (MDR is in spectral vicinity of QDs emission lines) Fig. S4(a), nonresonant quadrumer (MDR experience red shift from QDs emission spectrum) Fig. S4(d). We consider QDs as dipole sources with constant amplitude and do not take into account field distribution inside nanoparticles. For fixed QDs positions inside nanopillars we vary their emission wavelength and integrate PL intensity into different solid angles defined here as angle from the normal to substrate. For each spectral point

we normalize PL intensity to its value obtained for the case when the same number of dipoles are located inside unstructured GaAs film with the same height and integration is carried out for the corresponding solid angle. For resonant quadrumer five times overall PL enhancement is observed for $\pi/2$ solid angle (Fig. S4(b)) while for nonresonant oligomer there is no significant increase in the overall PL outcoupling efficiency (Fig. S4(e)). PL intensity enhancement at the border of the investigated spectral range can be explained by approaching the electric dipole Mie type resonance. The radiation pattern for nanostructure in Fig. S4(a) has a distinguished direction of the maximum dipole emission (Fig. S4(c)), while for QDs integrated in system Fig. S4(d)) it is difficult to distinguish special vectors of radiation propagation (Fig. S4(f)). However, it is worth mentioning that in both cases the most of the QDs emission power propagates towards the substrate, that can be avoided by optimizing quadrumers design, for example, using AlGaO layer as spacer between GaAs substrate and nanopillars. Isolated nanopillars also increase dipole source emission directivity, but the maximum intensity is 1.5 times less. In addition, field localization inside nanoparticles of the quadrumer is greater due to collective mode excitation through nanopillars local field interaction.

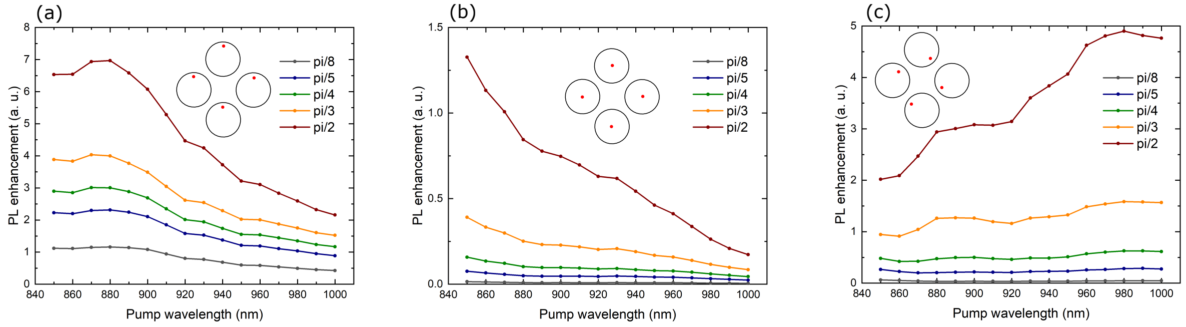

Figure S5: The dependence of the isolated quadrumer PL intensity from the dipole position inside nanopillars volumes integrated in various solid angles.

The QDs PL intensity enhancement is strongly sensitive to their position inside nanopillars. In Figure S5 results for three dipole source locations in xy-cross section of nanoparticles are presented. In all cases the presence of the oligomer increases the emission directivity, but spectral position of the maximum and its value changes. Spectral shift of 100 nm (between

cases described in Fig. S5(a) and S5(c)) and 5.4 times intensity variation (between cases described in Fig. S5(a) and S5(b)) can be achieved.

The second factor that strongly influence on QDs emission intensity concerns the amount of photons absorbed by QDs that is sensitive to the electric field localization factor in the volume of nanopillars ($L(\omega)$). Sample is illuminated by laser radiation with energy $J = N_0\hbar\omega$, where N_0 is number of photons. Some of these photons, $L(\omega)N_0$, will be absorbed in the semiconductor structure. Because the pump wavelengths used in the experiment corresponds to over-barrier excitation, then direct absorption of light by the QDs can be neglected compared to light absorption in the material of barrier layers (GaAs). As a result, $L(\omega)N_0$ electron-hole pairs will be generated in the barrier material and the part of them will be captured by QDs result in their excitation. Provided that the measurement time is much longer than the characteristic PL decay time, the pump power is weak and temperature is low, that is respected in the experiment, quantum yield of QD radiation is 100%. Thus emission intensity of the QDs embedded to the oligomer with greater absorption coefficients at the pump wavelengths will prevail over the others. We calculated absorption coefficients for two quadrumers differing in diameters and, as a consequence, the spectral positions of MDR. Mie-type mode of the smaller one ($d = 180$ nm) is in spectral vicinity of excitation wavelengths in our μ-PL experiment, the MDR of the other ($d = 180$ nm) has noticeable red shift (Fig. S6, colored area). Nanopillars' diameters slightly vary from that in the experiment because the modelling was conducted for the quadrumer without substrate for calculation simplification and given that we are interested in relative values. We obtained that the smaller oligomer has absorption coefficient peak at the wavelength of 750 nm (Fig. S6(a)), while the absorption value for the second structure there is almost half (Fig. S6(b)). For both cases it has blue shift from MDR, what has already been investigated in other works concerning all-dielectric nanophotonics.[1] The difference in absorption indices of the nanostructures coincides in order of magnitude with the delta in the PL intensity enhancement observed in the experiment for qualitatively the same difference in quadrumers MDR central

wavelengths.

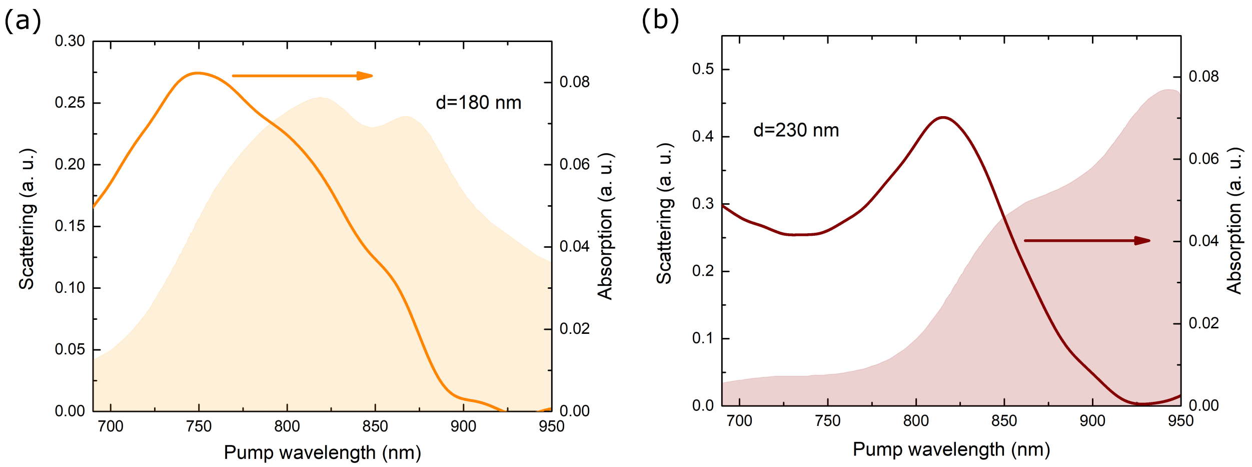

Figure S6: The numerically retrieved absorption (dashed curves) and scattering (colored area) spectra of GaAs quadrumer with height, $h = 300$ mn, interparticle distance, $s = 100$ nm and diameters, $d_1 = 180$ nm (a) and $d_2 = 230$ nm (b) located in the air without the GaAs substrate.

Table S1: Obtained PL enhancement

Case	Localization	PL directivity	Both
Experiment, LP	5	3	-
Calculation, LP	-	-	40
Calculation, LP	-	-	160

Section S2: Dark- ield spectroscopy Using Olympus microscope working in refraction regime we obtain dark-field images of several quadrumers and monomers (isolated nanopillars). The difference in the color of the structures indicates varying Mie-type modes structure (Fig. S7).

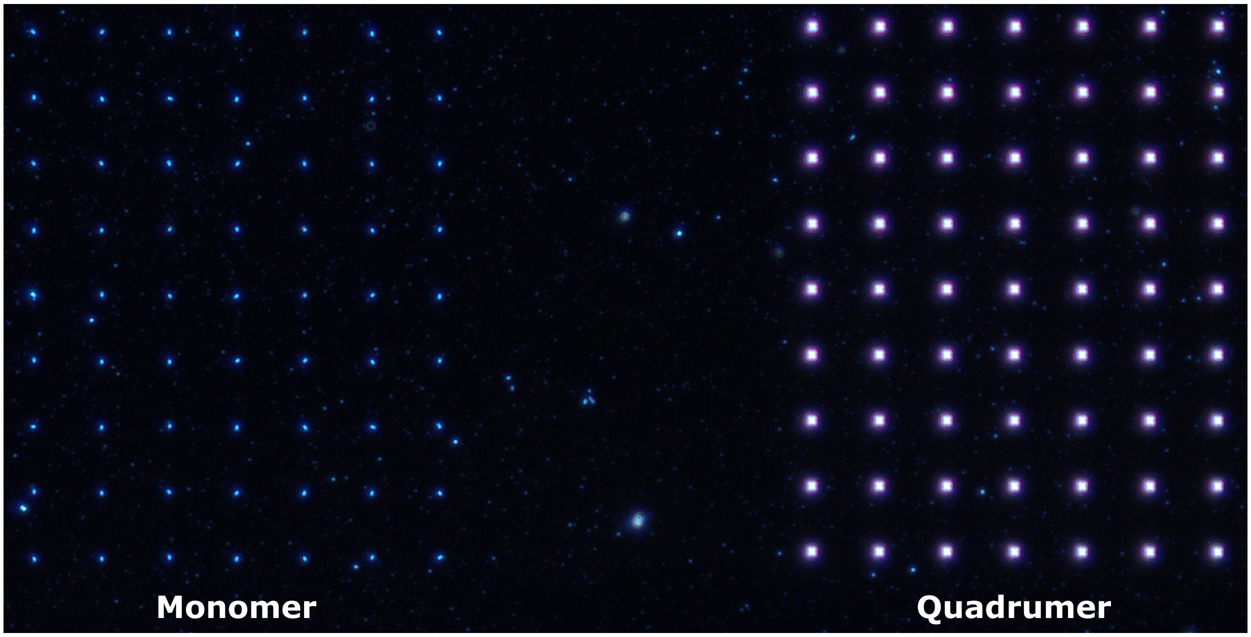

Figure S7: Dark field images of the samples under study. Left part–monomers, right part–quadrumers.

We conduct dark-field spectroscopy of isolated quadrumers in different arrays with the method described in the main part of the text (Fig. S8). With increased diameter the peak spectral position experiences red shift, that corresponds numerical predictions. The type of resonance is investigated numerically using the finite difference method in the time domain in Lumerical FDTD software. The geometry in the calculations repeats the experimental situation: the GaAs nanopillar on a GaAs substrate. The distribution of the electromagnetic field in the cross section of the nanopillar indicates the excitation of the MDR (Fig. S9).

We also calculated the emission spectra of various quadrumers using the finite element method in the software package COMSOL Multiphysics. A plane wave was used as pumping, and the scattering cross section of a single structure on the substrate was calculated. The results are presented in Fig. S10 for resonant sample in Fig. 2(b) in the main text with

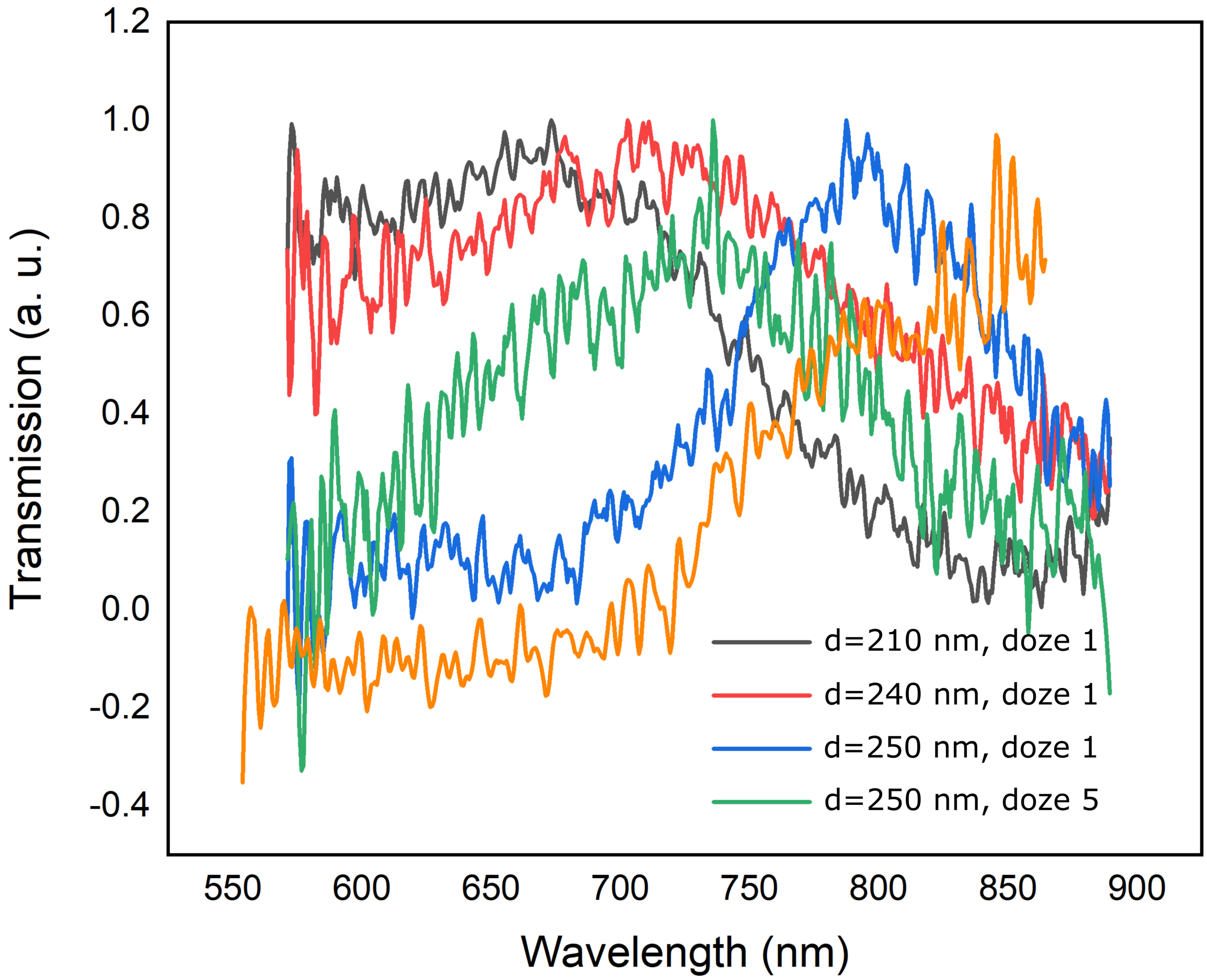

Figure S8: Dark field spectroscopy results for isolated quadrumers with various diameters. Its real value may be 15 nm shifted from the written one due to the inaccuracy of manufacturing technology. With increased doze the diamter of nanoparticles decreases approximately 10 nm per doze step.

h = 400 nm, spacing between nanoparticles of s = 100 nm, and d = 220 nm. When the radiation was decomposed into optical Mie-type modes in COMSOL, it was confirmed that in linear experiment in the main text precisely the MDR was measured.

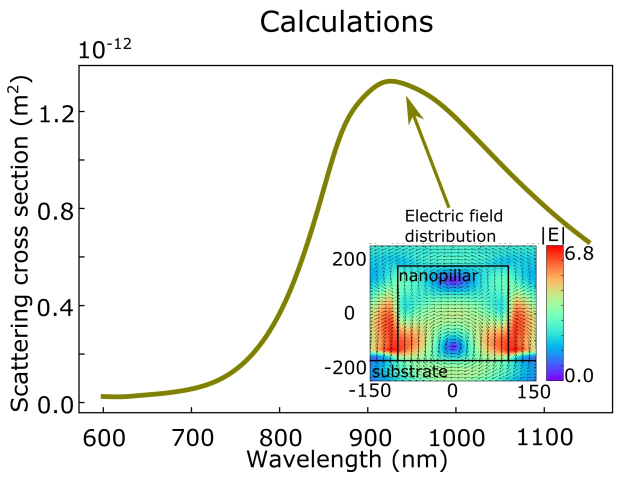

Figure S9: Numerical scattering cross section spectrum of quadrumer with resonance in the spectral range of QDs PL, obtained in Lumerical FDTD. Inset is local electric field distribution at the wavelength of 925 nm.

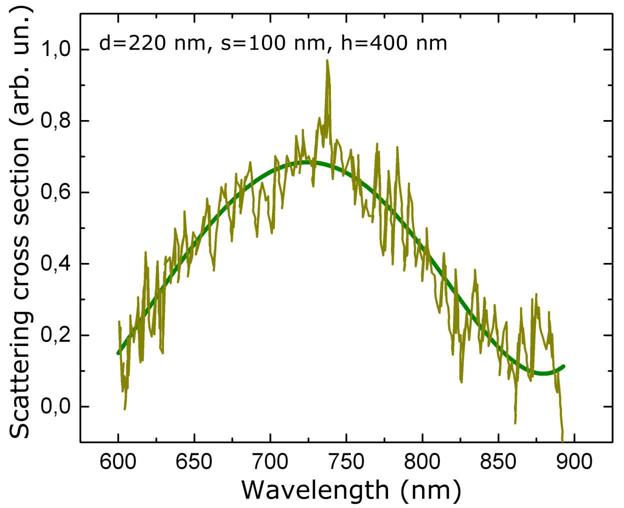

Figure S10: Numerical scattering cross section spectrum of the resonant quadrumer (obtained in COMSOL software) superimposed on the experimental scattering spectrum.

References

(1) Melik-Gaykazyan, E. V. et al. (2016). Third-harmonic generation from Mie-type resonances of isolated all-dielectric nanoparticles. Philosophical Transactions of the Royal Society A, **375**, 20160281.
